# The Occupational Health of Female Immigrant Caregivers: A Qualitative Approach

**DOI:** 10.3390/ijerph17217807

**Published:** 2020-10-25

**Authors:** Rocío de Diego-Cordero, Juan Vega-Escaño, Lorena Tarriño-Concejero, María Ángeles García-Carpintero-Muñoz

**Affiliations:** 1 Research Group CTS 969 Innovation in HealthCare and Social Determinants of Health, Faculty of Nursing, Physiotherapy and Podiatry, University of Seville, 41009 Seville, Spain; rdediego2@us.es; 2 Research Group CTS 1054 Interventions and Health Care, Red Cross, Spanish Red Cross Nursing School, University of Seville, 41009 Sevilla, Spain; 3 Research Group PAIDI-CTS 1050 Complex Care, Chronicity and Health Outcomes, Faculty of Nursing, Physiotherapy and Podiatry, University of Seville, 41009 Seville, Spain; agcarpin@us.es

**Keywords:** caregiver, immigrants, occupational health, working conditions, women, household services

## Abstract

In general, immigrants suffer poor working conditions. This is particularly true in the case of women, who constitute 48% of international migrants, and these poor conditions are closely linked to the sectors they mainly occupy, such as domestic and care-giving services. The aim of the present study was to investigate the working conditions of the female immigrant population living in southern Spain and how these conditions may affect their health. A qualitative study using semi-structured interviews and discussion groups was conducted over one year in 2019, with 61 immigrant women recruited. The sectors occupied by immigrant women were caregiving for dependent people and domestic services. Most of the female immigrants interviewed were working (63.94%), although the majority were employed in an irregular situation, with a very long working day. Among the main risks identified were biological risks, physical attacks, falls, wounds and musculoskeletal complaints related to handling patients and carrying out household chores. Most of them had not taken an occupational health test and did not report accidents occurring in the workplace for fear of losing their jobs. The main health problems were related to physical and mental health (such as musculoskeletal diseases and stress). These findings highlight the importance of making a major change in our perspective regarding the social value of including immigrant women in the labour market and the different aspects related to their health.

## 1. Introduction

Migration, defined by the International Organisation for Migration (IOM) as “the movement of a person or a group of persons, either across an international border, or within a State” [1] is an inevitable phenomenon which affects all countries. The goal of migrants has usually been to improve their standard of living. In recent years, the number of international migrants has increased significantly, reaching 272 million worldwide in 2019. Female migrants constituted 48 per cent of this international migrant stock [2].

In Spain, immigrants constitute 10.7% of the total population. In Andalusia, this figure is 7%, of which 49% are women [3]. As regards national origins, the flow of immigrants from South America to Andalusia increased from 2003 to 2008, and has decreased since 2009, with 7200 people registered in 2018 [4].

Immigrants are considered vulnerable people, due to the interaction of various factors such as their sociodemographic characteristics, capacities (knowledge, networks, access to resources and information, etc.), location and the impact of crisis-induced factors (separation, loss and lack of resources and opportunities or discrimination in access to assistance) [1]. Several studies have shown that the irregularity of work is a common feature in the situation of immigrant workers [5,6]. This population most commonly works in the so-called “3D jobs”: dangerous, dirty and demanding/degrading [7]. In comparison with the native population, immigrants usually perform low-skilled, temporary jobs, with low wages, greater physical requirements, inferior environmental conditions at work and greater exposure to occupational hazards [8,9,10], resulting in worse health outcomes and an increased risk of injuries and occupational diseases. Among the most common health problems among immigrant workers in Spain are stress, depression, sleep disorders, diffuse muscle pain, headaches and gastric complaints [11,12,13,14].

In European countries, the population is gradually aging, and there is a growing demand for care providers. Increasingly, care work is not provided by the native population, since it is not an attractive choice for workers who may have other work options [15,16,17]. Immigrant women therefore provide care in the domestic environment, and have assumed the traditional, well-defined characteristics of the caregiver in terms of invisibility, an unhealthy overload of work, deterioration in the quality of life and social relationships, physical fatigue and exposure to stressors [18,19,20]. To this, we must add the nature of their situation as migrants [17]. Previous studies have shown that the main health problems related to working conditions among female foreign domestic workers were physical, verbal, and sexual abuse, musculoskeletal strain, respiratory difficulties, mental health illness and infectious diseases, among others [21]. In addition, a recent bibliometric analysis of 21,457 documents concerning global migration health pointed out the need to investigate the health conditions of female migrant workers and promote research into migrant women’s health in general [22].

All of the above, the increase in immigrants who have arrived in Andalusia, their situation of vulnerability, the irregular jobs they perform, the negative health effects that this entails and the incorporation of this population into the care sector as a consequence of the aging population of European countries justify our research, whose objective has been to study the working conditions of the female immigrant population living in southern Spain and how these conditions may affect their health.

## 2. Materials and Methods

### 2.1. Design

The study had a qualitative descriptive design, an ethnographic approach. Authors such as Creswell (1998) [23], Alvárez-Gayou (2003) [24] and Mertens (2005) [25] indicated that this approach seeks to describe and understand phenomena from the perspective of each participant and collectively, based on the analysis of specific discourses and themes, as well as on the analysis of their possible meanings.

### 2.2. Sample

The study was carried out in five Non-Governmental Organisations (NGOs) in Andalusia which offer social and professional assistance to immigrants. The total number of informants was 61. The sample for the interviews (I) was made up of 43 women and the sample for the Focus Groups (FGs) consisted of 18 women.

Informants were included provided they were adult immigrant women living in Spain; were able to communicate (in fluent Spanish or English); were enrolled in NGOs; were working or had been working in companies that provide *home help services.*

The sampling was purposive and saturation criteria was used, and it was unlikely that new data, i.e., new categories or concepts, would be obtained in subsequent interviews [26].

### 2.3. Data Collection

Both interviews and FGs were carried out. The aim of the FGs was to deepen our knowledge of the aspects that emerged increasingly after the interviews touched on more profound topics and the motivating factors that were expressed only in the group dialogue, which allowed us to establish contextual interpretations for the registered responses [27].

Due to the participants’ lack of availability, the duration of both the interviews and the FGs ranged from 45 to 60 min. The interviews were carried out face-to-face on the NGOs’ premises (*n* = 38) and by phone (*n* = 5) in 2019, lasting 50 min. The FGs (*n* = 2) were carried out on the NGOs’ premises for 45–60 min. Both were audiotaped and transcribed verbatim.

The informants were selected at the NGOs where they received job advice. At the start of the training session, they were informed of the objectives of the study and invited to participate voluntarily. Those who agreed to take part were interviewed at the end of a training session about Occupational Health and Safety (OHS). The data collection process was completed following the principle of theoretical saturation [28].

A summative content analysis was carried out. After codification, the most significant units of analysis were extracted and the interrelationships between the different themes were identified. A final report was prepared with the statements from the interviews or FDs informants, indicated by “I-number” and years. The whole analytical process was carried out using the NVivo software.

### 2.4. Instruments

In 2006, the project called “Immigration, Work and Health” (ITSAL) began in Spain, from the collaboration of occupational health research groups located in different cities, whose general objective is to analyse the employment conditions and work of immigrant workers and their relationship with health. As specific objectives, the ITSAL project includes describing the personal and labour characteristics of migrant workers, knowing their perceptions about health, work and the relationships between them, describing their employment and work conditions, their knowledge about occupational risks and their health protection rights at work, and analyse the relationships of all these elements with indicators of physical, mental and social health [8]. The topic guide was based on this project. Another topic guide was written for the FGs.

A group of experts in occupational health (n = 7), migration (n = 6) and women’s health (n = 5) carried out an analysis of the content of these scripts to assess their suitability between May and July 2018. Two female users of the NGO were also consulted to show their degree of agreement with, and understanding of, the topic guide, and their contributions were also taken into account. Expert input on the topic guides was received and amendments were made accordingly (Table 1).

### 2.5. Data Analysis

The study took a phenomenological perspective, considering and relating to human beings in connection with their world [24]. To achieve this, two researchers read through all the field notes and interview transcriptions several times, to gain an overall understanding of the content. Likewise, the other two authors read samples of the field notes and interviews. The analysis was carried out by organizing descriptive labels, focusing on emerging or persistent concepts and similarities/differences in the participants’ behaviour and statements. Three main themes reflected the categories: “Reasons/motivating factors for migration”, “Working women” and “Occupational health safety”.

### 2.6. Validity

This research followed the criteria of the Consolidated Criteria for Reporting Qualitative Studies (COREQ) [29]. Aguilar-Gavira and Barroso-Osuna (2015) [30] proposed that studies should use five different methods of triangulation: sources, researchers, theoretical, methodological and multiple. In our study, we used methodological triangulation, which enabled us to test the level of consistency and to resolve discrepancies (see the Supplementary Material, Table S1).

The methods used to improve the validity of this qualitative study included data triangulation (including participants with different sociodemographic characteristics) and triangulation of data analysis via different researchers (belonging to the group of experts who analysed the content of the topic guides).

### 2.7. Ethical Considerations

All the women included in the project voluntarily agreed to participate. They received verbal and written information about the study, as well as a letter of confidentiality signed by the research team before the interviews and FGs. The information included the potential risks of the study, voluntary participation, and explanations about the right to refuse to answer questions or terminate interviews at any point. In addition, the participants were informed about the interviews and FGs, that they would be recorded on audio and quoted anonymously in publications, and that all personal identifying information would remain blinded. In addition, verbal consent was obtained from participants. The study was approved by the Andalusian Research Ethics Committee, Spain (Code: 0731-N-19).

### 2.8. Reflexivity

The interviews were conducted by academic researchers, who collaborate with NGOs as voluntary staff. To avoid power imbalances, the interviews and FGs were carried out on the NGOs’ premises, as these were familiar territories for the participants and where they felt free to express themselves.

## 3. Results

### 3.1. Informants

The total sample consisted of 61 female Latin-American immigrants (43 interviewees and 18 FGs participants), with an average age of 41.30 (SD = 10.36), who had lived in Spain for 5.52 (SD = 6.33) years. The educational level of the participants was 32.78% with high school studies and 27.86% with university studies (Table 2).

### 3.2. Reasons/Motivating Factors for Migration

The main motivating factor behind migration was to escape from precarious socioeconomic conditions verging on poverty and to find a job to be able to enjoy a better standard of living, principally for their families. Nearly all the participants have children back home to whom they send payments for their upkeep and to help them out: this, in fact, was their principal reason for migrating. In total, 95% of the participants commented:

“In my country, poverty is everywhere, and there are no opportunities for anyone to achieve something in life” I7 (49 years).

“I do it to get a better quality of life and help my son who is in my country (Ecuador).” I42 (50 years).

“To ensure for my daughter and myself a better future with economic stability” I43 (37 years).

“There (home country) it was difficult to find work, and the elderly are not cared for as much as here” I19 (41 years).

“I came here to make a new life and I have a daughter in Colombia who I’m supporting” I25 (58 years).

“(...) because if I´m here I can give my family a better quality of life than I can give in my country” I7, FG1 (35 years).

Another major reason was public safety and the fact that crime here was practically non-existent compared with their countries of origin. Some motives for political asylum described by 50% of the participants were also detected.

“It’s not the same there-here, you are completely safe” I1, FG2 (50 years).

“I came because of the difficult situation in my country: there’s a civil war” I2 (37 years).

In addition, the Spanish language and the cultural similarities with Andalusia were seen as a prime reason for choosing their destination, in comparison with other factors such as economic development and job opportunities in other parts of Spain or wealthier European countries. In total, 90% of the participants remarked.

“What’s more, most people come here because of the language, because you can move around, but language is also a problem for us, because none of us can speak English or French” I2, FG2 (62 years).

“The Andalusian people are very like us” [...] “cheerful and home-loving” [...] “we can get on better with an Andalusian than with a Catalan” I1, FG1 (24 years).

### 3.3. Working Female Immigrants

Most of our interviewees were employed (63.94%), but their contractual situation was unstable, and they worked long hours.

“I work part-time” [...] “once a week, but they don’t give me a contract” I2, FG2 (62 years).

“I worked as a child-minder in a house, but I wasn’t registered with the social security” I38 (43 years).

“I (work) 24 h a day 7 days a week, and don’t ask for one weekend a month like other girls because there’s no-one else who’ll stay here. So, if you want to earn your monthly salary, you have to accept the conditions, which are 24/7” I7, FG1 (35 years old).

The main professional sector was domestic service and caregiving for dependent people, such as children, and the elderly with irreversible cognitive disorders such as dementia. All of the participants told us:

“I look after a senior citizen, to help her do her things like cooking, cleaning” [...] “everything” I5 (37 years).

“I’ve worked as a live-in assistant taking care of elderly people with Alzheimer’s, senile dementia, diabetes” I8, FG1 (50 years).

“I’ve worked with an older person with Alzheimer’s disease” I4, FG1 (27 years).

“I’ve worked as a caregiver” I8 (51 years).

“I’ve had two jobs since I arrived here - I ‘ve cared for older people” I7, FG1 (35 years).

As regards the tasks performed, 55 participants did far more work than was required of them and more than they were professionally trained to do, often carrying out the roles of health carers or nurses. In total, 30% of the participants lived in rural areas, the domestic tasks even included looking after crops and livestock: these jobs usually require a huge physical effort and involve long working hours. They commented:

“When I look after the elderly, I do the kitchen, the toilet, the house, all the general cleaning, as well as changing the dressings on their sores” I10 (55 years).

“I cooked, ironed, cleaned, went shopping, controlled all the medication for the person I was looking after, such as injecting insulin, putting on patches and measuring blood pressure, as well as looking after their diet, movement and personal hygiene” I41 (43 years).

“Doing the domestic chores, cleaning, washing (...) I also helped them to pick oranges and took care of the animals” I7 (49 years).

“(…) to care for her, help her to do the housework such as cooking or cleaning, among others” I5 (37 years).

“To take care of the elderly and children” I17 (29 years).

“I did all the housework and I also took care of an older person” I22 (46 years).

“Everything related to work at home (...) in addition, I looked after older people with reduced mobility” I20 (48 years).

“Practically all housework tasks” I39 (48 years).

As for the occupational risks that they felt they were exposed to, one of the most dangerous biological risks that was mentioned by 85% of the participants was the risk of infectious disease due to contact with the person they were looking after. However, another risk was identified by 24 participants. They mentioned:

“When caring for elderly people, showering them without gloves” I4 (45 years).

“Every year I get the flu” [...] “it’s inevitable when you work looking after dependent people” I30 (48 years).

“Others are the spread of the patient’s diseases, such as the flu” I23 (49 years).

“Risks of slipping over” I12 (34 years).

“I can cut myself at home with a knife or slip” I10 (55 years)

“I hit my knee on the patient’s winch. These kinds of blows are normal when taking care of him” I21 (54 years).

Nineteen participants also mentioned the risk of being exposed to chemicals in the cleaning products they work with.

“I used ammonia and had problems with it because it gave me a headache” I19 (41 years).

“There’s a degreaser that is very strong” I17 (29 years).

“In the kitchen with chemical cleaning products” I25 (58 years).

Other risks detected included injuries after being assaulted by patients with mental disorders, as well as bruises and injuries from accidents and falls. Osteoarticular injuries caused by moving the patients were also frequent, and in all cases, they stressed the lack of personal protective equipment and the workplace not being ergonomically adapted.

“Some Alzheimer’s patients have hit me—there’s the risk of being attacked” I6 (49 years).

“I fell out of the window on the first floor in the home of a person I was looking after.…” I26 (43 years).

“I worked 25 months without a contract and was exposed to too many risks because I took care of a woman who weighed over 150 kg. Every day, I had to lift her out of bed and that was terrible for my back” I27 (42 years).

In total, 87% of the participants had received no training in Occupational Health and Safety (OHS) and seven participants who admitted they had had some training said that it was insufficient.

“The only training, I had was to browse and read things online” I8 (51 years).

“They never train you; they’ve never done it with anyone I know” I16 (49 years).

“Once I wanted to participate in a training programme that lasted two days, but I had to travel and finally I wasn’t able to do it” I12 (34 years).

### 3.4. Occupational Health Safety

Over half the women had never had an occupational health examination. In addition, they mistook the occupational health examination for a general medical check-up.

“As I have my own insurance, I was going to have the check-up done on my own” I15 (59 years).

“I had a full health test and they asked me about diseases” I2 (37 years).

“I take care of my health myself, because I have my public health insurance, and I usually go when necessary. I had my last check-up a year ago” I8 (51 years).

The main health problems detected relate to both physical and mental health.

“(…) when you are working with older people, it affects your physical and mental health” I8, DG1 (50 years).

Among them, the most common problems among those interviewed were related to stress, as well as osteoarticular and muscular pain in both halves of the body (upper and lower). In mental health, stress problems due to work overload were the common.

“If as well as caring for an elderly person, the family also tells you that you have to clean the house and look after pets, then your stress levels increase” I13 (49 years).

“(...) the fatigue is physical, but you also get mental fatigue at night” I1, FG1 (24 years).

“(…) very tired since it was hard to bend down to do the cleaning and my feet and back hurt a lot.” I12 (34 years).

In physical health, the interviewees commented on osteoarticular and musculoskeletal pain, also related to work overload.

“My bones and joints ache (...) it doesn’t matter who you work for, you always work long hours” I25 (58 years).

“In my case, my back is the worst bit, I don’t stop (...) and when I get to the end of the day, my feet feel sore” I10, FG1, (49 years).

Many known cases of occupational accidents were not reported or not identified:

“I fell and had a bruise on my foot and inflammation for 20 days, but I didn’t go to the doctor” I20 (48 years).

“I’ve suffered superficial wounds, but they weren’t serious, and I’ve also banged my leg against the corner of the table or cut myself with knife when cooking.” I10 (55 years).

“I had a contract, but I didn’t phone her children—which was stupid of me” [...] “she had a fall at four o’clock in the morning and I lifted her up myself and carried her to bed—and then I got the hernia” I2, FG2 (62 years).

“I cut my fingers with the meat slicer” I31 (30 years).

“I climbed a tree to pick some oranges and fell and scratched my eye” I24 (27 years).

When they had a health problem, most of the women interviewed turned to the traditional natural medicine they used in their home country and/or self-medicate. Eleven participants visited the health services when they needed medical attention or were very ill.

“I started to make my tea, which was celery tea, by the way, and I made another cup of parsley tea to ease the pain” I7, FG1 (35 years).

“I self-medicate. My recipe is ginger with lemon and cinnamon, in very hot tea, twice a day, together with paracetamol … and well, I’m better now” I10, FG1 (49 years).

“Algae, tea to reduce stomach pain” I1, FG1 (24 years).

“For the cold I usually drink hot milk with honey, hot milk with thyme, honey and lemon (...) the natural recipes of my country. (…) I only visit to the doctor when I’m very ill” I2, FG2 (62 years).

## 4. Discussion

Our findings show that the main reasons for immigrants to migrate to Spain and, in particular, to Andalusia are to look for a job to improve their standard of living, to send foreign currency home to support their families, and linguistic and cultural proximity. Immigrant working women are employed irregularly, and under working conditions which demand long hours, as well as a lack of individual protection equipment and no information on occupational risk prevention. The main risks identified are the risk of biological infection, falls, blows and injuries. Occupational accidents are also rarely reported. The main health problems include musculoskeletal disorders, which accompany problems of physical exhaustion and mental health. The underuse of occupational health resources is also reflected when dealing with problems, with the participants not attending occupational health examinations and opting in most cases for traditional medicine.

As regards the reasons/motivation factors for migration, the ultimate objective of immigrant women is to enter the labour market to improve their standard of living and the economic situation of their family back home [11]. This was confirmed in our study, where the participants migrated to Andalusia for the same reasons mentioned above. Migration can be considered as a social elevator for the families of these women in their home country: by sending home foreign currency, they improve their socioeconomic status [31,32]. Many of the women in our study had been to university but carried out unskilled jobs with long working hours and in conditions of irregular employment. This has been corroborated in a number of qualitative studies, which show that the immigrant population has to put up with precarious working conditions [11,12,13,33,34,35].

As regards working immigrant women, the situations found in our study relate to the sector occupied mainly by these women, domestic service, which according to the International Labour Organisation (ILO) [36], is mainly carried out by women. Domestic work and care within the home is regulated by law in Spain in Royal Decree 1620/2011, but despite this, many of these immigrant women are in an irregular situation [37]. However, changes in the population pyramid are taking place in the European countries, whose population is aging. The expected dependency ratio in Spain in 2029 will be 59.2%, and life expectancy will rise to 84 years for men and 88.7 years for women. The percentage of the population over 65 will double in the next four decades [38], and the number of elderly people living alone will increase, so there will be an even greater demand for care [16]. According to the 2015 ILO report [39], most domestic workers are international immigrants who work irregularly in private homes, a circumstance which makes it more likely that their working documentation is in order. A transmission chain of domestic work and care can therefore be established, which operates with the categories of gender, social class and ethnicity, from Spanish women with a medium or high socioeconomic level to immigrant women who, due to their particular characteristics, represent a cheap, relatively well-qualified work force [32,40].

This idea emerges from our interviewees’ comments, where we observe that the gender mandate assigned to care operates exclusively through women, generating global care chains which are determined by three important aspects: the predominance of private, commercial or family services; the lack of public services; the central role played by women as the articulating axis of the entire network [41,42,43,44].

In the case of Latin-American immigrant women, they also have a closer cultural affinity with the language, religion and value system, among other factors, thus representing a more suitable profile than women of African, Asian or Eastern European origin, and they are therefore preferred by employers for domestic work and caregiving. Evidently, caregiving not only includes the job itself, but also involves establishing a relationship with the person cared for and in most cases with the family receiving the care [16,45].

As regards occupational health safety, previous studies have shown that immigrant workers in Spain had higher disease presenteeism and lower disability rates than the native population [46]. Work is the main origin of health problems among the immigrant population, but the figures for presenteeism are high [47,48,49], as is self-perceived health, since the highest priority is their personal economic sustainability and that of the family unit [50], among other reasons, as shown in this study. Furthermore, again in agreement with the results of our study, the immigrant population do not generally feel they have enough legal support to stand up for their rights and for better working conditions [30,38]. To this, we must add one important issue: the cultural differences related to their perception of health/illness and their relation to work [51]. Here, there is evidence of the Healthy Immigrant Effect (HIE). The HIE implies that immigrants who have recently arrived at their country of destination have a better health status compared to the national population with similar sociodemographic characteristics, which will eventually converge to the level of the native population the longer they stay [51,52].

Immigrant women in Spain corroborate these data, where it is evident that domestic and care work provide many immigrants with job stability and the possibility of obtaining a residence permit, which results in a better standard of living, although their main health problems are caused by their working conditions [53].

In relation to the occupational health test, our results show that very few were taken, despite the fact that Spanish legislation on the prevention of occupational risk also applies to the sector of the home help services companies in which our interviewees are employed. In the case of Spain, the labour sector for caregiving of dependent people is regulated by Law 39/2006, of December 14th, on the Promotion of Personal Autonomy and Caregiving for people in a situation of dependency, which establishes the rights of all people to be cared for under equal conditions. This law regulates the working conditions of people dedicated to the care of dependent people, with modifications established in the different labour agreements at the provincial level. However, all the agreements recognise the Law 31/1995 of November 8th, on the Prevention of Occupational Risks, as the applicable norm to protect workers [54], which recognises the obligation to monitor workers’ health, including the occupational health test.

Finally, some recommendations linked to results are aimed at evaluating the caregiving, highlighting that in its nature it is a qualified, improvable job, which has been termed “care gain” [55] by which the host country benefits from the caregiving skills of the immigrant workers; others aim to reformulate the regulations and policies to prevent a social transformation that may work to the detriment of immigrant caregivers [43].

Another aspect to be discussed is the mistreatment suffered by immigrant caregivers, which is dealt with in previous studies [56], although in our study we did not detect this phenomenon after analysing the interviewees’ comments.

This study had certain limitations. To infer our results to the immigrant community in Andalusia or in Spain is particularly difficult because a convenience sample was used. Furthermore, only a Latin American population was studied, leaving out other ethnic groups which make up the full immigrant community. In addition, the sample was collected through NGOs and the reality of immigrant caregivers who do go to NGOs is different from the reality of immigrant caregivers who do not go to NGOs for two mean reasons: on the one hand, NGOs collaborate as mediating agents for the use of social services, making them more accessible to the immigrant community, and on the other hand, they provide services such as training and professional guidance, which places study participants in an advantageous situation. Nevertheless, the study also has some strengths. The gender-based, cultural analysis of the working conditions of immigrants in Spain showed the women’s vulnerability, which has become more acute in situations such as the COVID-19 pandemic. In addition, women are more likely to be infected with the virus, given their predominant role as caregivers and as front-line health care providers [57].

## 5. Conclusions

The inclusion of migrant women in the labour market has socioeconomic implications and brings a two-fold benefit: on the one hand, it is a social elevator for the women’s families back home, as they send back foreign currency which helps to support the family and improve their finances; on the other, the incorporation of these women into the domestic and care-giving sector creates added value in the receiving country, since it allows well-qualified women from that country to enter the labour market by delegating their domestic work to the immigrant women. This research shows that what needs to change on a social level is our viewpoint, with a positive approach towards these immigrant women who join the service and care sector, since they represent an important added value for the host country, shown in the benefits of the local population who receive the care.

Despite the fact that working conditions (irregularity, overload) are the main cause of the health problems reported by immigrant women, it is the economic sustainability of their families that constitutes the main factor behind the high rates of presenteeism, the under-reporting of work accidents, the high levels of self-assessed health and self-medication, and the lack of use of public health services.

## Figures and Tables

**Table 1:** Topic guide.

Interview Topic Guide
Sociodemographic characteristics
Age, country of origin, level of education
Reason for Migration: Why did you decide to come to Spain?
Time and characteristics of residence in Spain (Who do you live with? Number of children/relatives).
Semi-structured themes
Socio-labour characteristics
Since arriving in Spain, which sectors have you worked in? What tasks have you been doing?
Working conditions
Current employment ^1^ situation (in search of employment, unemployed, employed, social insurance and regular work, type of work and functions performed).
Daily working conditions (weekly schedule, overtime, work of day: complete/partial or otherwise).
If unemployed, previous work history (in Spain).
Exposure to occupational risk (identification, attributed severity).
Types of risks (handling of vehicles, machinery or other dangerous tools, use of chemical products).
Training in OHS by the company (characteristics, type of training, duration).
Occupational health
Occupational accidents throughout working life in Spain (description, type of accident, severity, required health care, sequelae or injuries, impact on job).
Occupational Health Exam (frequency, characteristics and tests performed).
Sick leave (reason, duration, impact on job).
Current health status (perception of health, presence of chronic problems).
Association of health problems with work activity (new symptoms, worsening disease)
Vaccination at the workplace.
**FGs topic guide**
Sociodemographic characteristics
Age, country of origin, level of education
Migratory aspects:
*Why did you decide to come to Spain?*
*Is Spain the final destination or only a transit before moving to another European country?*
*Reason for coming to or staying in Spain (language, support network of relatives or other compatriots). Reason for choosing Andalusia*
*Expectations or previous knowledge about Spain*
Time and characteristics of residence in Spain
*Who do you live with? (Number of children / relatives).*
*Family income.*
*Co-responsibility between men and women in domestic tasks*
*Family care: children in country of origin, percentage of salary sent to help with their maintenance*
Working conditions
Current employment ^2^ situation (in search of employment, unemployed, employed, social insurance and regular work, type of work and functions performed).
Working daily conditions (weekly schedule, overtime, shift work, working day: full-/part-time or other).
Occupational health
Occupational accidents throughout working life in Spain (description, type of accident, severity, required health care, sequelae or injuries, labour impact).
Occupational Health Exam (frequency, characteristics and tests performed).
Labour leave (reason, duration, labour impact).
Current health status (perception of health, presence of chronic problems).
Association of health problems with work activity (new symptoms, worsening disease)
Vaccination at the workplace.
Actions taken when they become ill (self-medication, help from friends or neighbours, alternative medicine, etc.)

Note: ^1,2^ work or business; occupation.

**Table 2:** Sociodemographic characteristics and employment status.

Variable	Participants (*n* = 61) Mean (SD)/[%]		
	Interviews (*n* = 43)	Focus Groups (*n* = 18)	Total Participants (*n* = 61)
Age (years)	38.2 (12.45)	44.4 (13.18)	41.3 (10.36)
Country of Origin			
Argentina	[0]	[5.75]	[1.64]
Bolivia	[9.3]	[11.1]	[9.84]
Chile	[2.3]	[0]	[1.64]
Colombia	[11.62]	[11.1]	[11.48]
Ecuador	[2.3]	[0]	[1.64]
El Salvador	[4.5]	[0]	[3.27]
Guatemala	[2.3]	[0]	[1.64]
Honduras	[11.62]	[11.1]	[9.84]
Nicaragua	[37.5]	[22.4]	[32.78]
Peru	[6.9]	[22.4]	[13.11]
Dominican Republic	[9.3]	[0]	[6.56]
Venezuela	[2.3]	[16.7]	[6.56]
Residence in Spain (years)	4.26 (5.52)	6.78 (14.12)	5.52 (6.33)
Number of children	1.97 (1.2)	1.27 (0.78)	1.62 (1.41)
Graduate Course			
Primary school	[18.6]	[16.7]	[18.03]
Secondary school	[25.7]	[11.1]	[21.33]
Bachelor	[41.8]	[16.7]	[32.78]
University studies	[13.9]	[55.5]	[27.86]
Employment			
Yes	[72.38]	[55.5]	[63.94]
No	[27.62]	[44.5]	[36.06]

## Supplementary Materials

The following material is available online at https://www.mdpi.com/1660-4601/17/21/7807/s1: Table S1: Consolidated criteria for reporting qualitative studies (COREQ): 32-item checklist.
