# Supplementary material for: The Occupational Health of Female Immigrant Caregivers: A Qualitative Approach
Source: Int J Environ Res Public Health. 2020 Oct 25;17(21):7807. doi: 10.3390/ijerph17217807 (PMC7663160; doi:10.3390/ijerph17217807)
Supplement: Supplementary file 1 [file ijerph-17-07807-s001.pdf]

Table S1. Consolidated criteria for reporting qualitative studies (COREQ): 32-item checklist

No	Item	Guide questions/description	Response
Domain 1: Research team and reflexivity			
Personal Characteristics			
1.	Interviewer/facilitator	Which author/s conducted the interview or focus group?	All the interviews were conducted by three authors: Author C, Autor B and Author A.
2.	Credentials	What were the researcher's credentials? E.g. PhD, MD	Author D and Author A had a PhD. Author C and Author B had an MSc (Health Sciences). In addition, Author A and Author B were OHNs.
3.	Occupation	What was their occupation at the time of the study?	All authors were research professors. In addition, Author B was working as an occupational health nurse.
4.	Gender	Was the researcher male or female?	Author D, Author A and Author C were female and Author B was male.
5.	Experience and training	What experience or training did the researcher have?	All researchers had experience in carrying out qualitative research. Author C, Author A and Author B had been trained to conduct interviews and Author A and Author D had training in social research.
Relationship with participants			
6.	Relationship established	Was a relationship established prior to study commencement?	No.
7.	Participant knowledge of the interviewer	What did the participants know about the researcher? e.g. personal goals, reasons for doing the research	Name, occupation, reasons for carrying out the research.
8.	Interviewer characteristics	What characteristics were reported about the interviewer/facilitator? e.g. Bias, assumptions, reasons and interests in the research topic	Name, occupation, contact method, reasons for carrying out the research.
Domain 2: Study design			

Theoretical framework			
9.	Methodological orientation and Theory	What methodological orientation was stated to underpin the study? e.g. grounded theory, discourse analysis, ethnography, phenomenology, content analysis	A phenomenological and ethnographic approach, with discourse and content analysis.
Participant selection			
10.	Sampling	How were participants selected? e.g. purposive, convenience, consecutive, snowball	Convenience sampling and snowball sampling.
11.	Method of approach	How were participants approached? e.g. face-to-face, telephone, mail, email	Face-to-face and by phone.
12.	Sample size	How many participants were in the study?	61 immigrant women living in the south of Spain.
13.	Non-participation	How many people refused to participate or dropped out? Reasons?	17, for work reasons (mainly lack of time) and other personal reasons.
Setting			
14.	Setting of data collection	Where was the data collected? e.g. home, clinic, workplace	The interviews were carried out on the NGO's premises, also in other places agreed with the participants to facilitate participation. Phone calls were also used when participants requested this.
15.	Presence of non-participants	Was anyone else present besides the participants and researchers?	In all the NGOs, there were specialists who could offer social-labour orientation.
16.	Description of sample	What are the important characteristics of the sample? e.g. demographic data, date	Immigrant women who had completed an OHS training course of at least 20 hours.
Data collection			
17.	Interview guide	Were questions, prompts, guides provided by the authors? Was it pilot tested?	Yes. / Yes.
18.	Repeat interviews	Were repeat interviews carried out? If yes, how many?	No.
19.	Audio/visual recording	Did the research use audio or visual recording to collect the data?	Audio recording.

20.	Field notes	Were field notes made during and/or after the interview or focus group?	During
21.	Duration	What was the duration of the interviews or focus group?	Average 50 minutes (interview) and 45-60 minutes (focus groups).
22.	Data saturation	Was data saturation discussed?	Yes.
23.	Transcripts returned	Were transcripts returned to participants for comment and/or correction?	They were reviewed by 2 informants.
Doman 3: Analysis and findings			
Data analysis			
24.	Number of data coders	How many data coders coded the data?	Four (Authors A, B, C D).
25.	Description of the coding tree	Did authors provide a description of the coding tree?	Yes.
26.	Derivation of themes	Were themes identified in advance or derived from the data?	Themes were derived using both methods.
27.	Software	What software, if applicable, was used to manage the data?	NUD*IST Nvivo 12.
28.	Participant checking	Did participants provide feedback on the findings?	They were reviewed by 2 informants.
Reporting			
29.	Quotations presented	Were participant quotations presented to illustrate the themes/findings? Was each quotation identified? e.g. participant number	Yes. / Yes.
30.	Data and findings consistent	Was there consistency between the data presented and the findings?	Yes.
31.	Clarity of major themes	Were major themes clearly presented in the findings?	Yes.
32.	Clarity of minor themes	Is there a description of diverse cases or discussion of minor themes?	Yes.
